# Bone Marrow Stem Cells Expressing Keratinocyte Growth Factor via an Inducible Lentivirus Protects against Bleomycin-Induced Pulmonary Fibrosis

**DOI:** 10.1371/journal.pone.0008013

**Published:** 2009-11-24

**Authors:** Susana Aguilar, Chris J. Scotton, Katrina McNulty, Emma Nye, Gordon Stamp, Geoff Laurent, Dominique Bonnet, Sam M. Janes

**Affiliations:** 1 Centre for Respiratory Research, Rayne Institute, University College London, London, United Kingdom; 2 Hematopoietic Stem Cell Laboratory, London Research Institute, Cancer Research UK, London, United Kingdom; 3 Experimental Pathology Laboratory, London Research Institute, Cancer Research UK, London, United Kingdom; 4 Department of Histopathology, Imperial College London, London, United Kingdom; University of Giessen Lung Center, Germany

## Abstract

Many common diseases of the gas exchange surface of the lung have no specific treatment but cause serious morbidity and mortality. Idiopathic Pulmonary Fibrosis (IPF) is characterized by alveolar epithelial cell injury, interstitial inflammation, fibroblast proliferation and collagen accumulation within the lung parenchyma. Keratinocyte Growth Factor (KGF, also known as FGF-7) is a critical mediator of pulmonary epithelial repair through stimulation of epithelial cell proliferation. During repair, the lung not only uses resident cells after injury but also recruits circulating bone marrow-derived cells (BMDC). Several groups have used Mesenchymal Stromal Cells (MSCs) as therapeutic vectors, but little is known about the potential of Hematopoietic Stem cells (HSCs). Using an inducible lentiviral vector (Tet-On) expressing KGF, we were able to efficiently transduce both MSCs and HSCs, and demonstrated that KGF expression is induced in a regulated manner both *in vitro* and *in vivo*. We used the *in vivo* bleomycin-induced lung fibrosis model to assess the potential therapeutic effect of MSCs and HSCs. While both populations reduced the collagen accumulation associated with bleomycin-induced lung fibrosis, only transplantation of transduced HSCs greatly attenuated the histological damage. Using double immunohistochemistry, we show that the reduced lung damage likely occurs through endogenous type II pneumocyte proliferation induced by KGF. Taken together, our data indicates that bone marrow transplantation of lentivirus-transduced HSCs can attenuate lung damage, and shows for the first time the potential of using an inducible Tet-On system for cell based gene therapy in the lung.

## Introduction

Many common diseases of the gas exchange surface of the lung have no specific treatment but cause serious morbidity and mortality. Injury to the lung parenchyma may result in a spectrum of diseases from the acute respiratory distress syndrome (ARDS) to idiopathic pulmonary fibrosis (IPF) [1]. Histologically, IPF is characterized by alveolar epithelial cell injury/activation, interstitial inflammation, fibroblast proliferation and exaggerated accumulation of collagen and extracellular matrix in the parenchyma, leading to irreversible loss of lung function. Animal models are widely used to study the evolution of such fibrotic responses, and the intra-tracheal administration of bleomycin is the best-characterized experimental model of lung fibrosis [2], [3], [4]. Despite its intrinsic limitations, the bleomycin model shares many clinical features with the human disease [5], [6]. Bleomycin-induced lung fibrosis is associated with an increase in classical pro-inflammatory cytokines including TNF-α, IL-1β and IL-6 followed by a switch to pro-fibrotic markers (TGF-β1, fibronectin and pro-collagen-1) [1], [7]. Epithelial cell apoptosis makes a significant contribution to the disease but type II pneumocytes appear to function as local stem cells, and functional recovery depends on their proliferation to restore the normal alveolar architecture [7].

Bone marrow-derived stem cells (BMDC) are being evaluated for their therapeutic potential in several lung diseases including pulmonary fibrosis [8], [9], [10]. They can both engraft in injured lung [8], [10], [11], [12] and in some cases are thought to acquire epithelial characteristics [8], [12], [13]. Within the adult bone marrow stem cells, there are two main populations: the hematopoietic stem cells (HSCs) and the mesenchymal stromal cells (MSCs). It has been described that MSCs have the ability to differentiate into multiple cell types *in vitro* [14], [15], [16]. MSCs improve outcome in the bleomycin model, possibly through immunosuppression and alteration of the cytokine milieu in the inflammatory stage of the disease process [8], [17], [18]. MSCs are an interesting therapeutic tool as it is suggested that they are immuno-privileged, which may allow their use for allo-transplantation therapies [10], [19], [20], [21], [22], [23], [24]. On the other hand, HSCs are recognized as the main source of adult stem cells having the ability to self-renew and to differentiate into all blood lineages. HSCs are also progenitors for several cell types, including endothelial [25] and epithelial [26], myocytes [27] and neurons [28]. In the lung, some groups have shown that HSCs are able to become alveolar type II lung cells after bone marrow transplantation [11], [13], [29]. After lung injury, the contribution of BMDC to lung repair has been reported in different injury models, such as Lipo-polysaccharide-induced lung injury [30], cystic fibrosis [31], radiation pneumonitis [29] and asbestos-induced pulmonary fibrosis [32]. Nevertheless, very little is known about the potential of HSCs in bleomycin-induced lung fibrosis, of serving as useful vehicles to express genes that may alter the pulmonary epithelium, regardless of their ability to differentiate into lung cell types.

Keratinocyte growth factor (KGF), also known as FGF-7, is a member of the fibroblast growth factor (FGF) family, and acts specifically on epithelial cells [33]. KGF is a potent mitogenic factor in a variety of epithelial cells including keratinocytes [34], as well as mammary [35] and pancreatic ductal epithelia [36]. KGF stimulates type II cell proliferation *in vitro* [37], and increases the expression of surfactant proteins in cultured alveolar type II cells [38]. Administration of exogenous rhKGF ameliorates lung injury in a variety of animal models. KGF pre-treatment reduces lung injury induced by acid instillation [38], bleomycin instillation [39] and α-naphthylthiourea [40]. In the bleomycin model, KGF improves both morphological damage to the alveolar epithelium and inflammation [39]. Hence this cytokine is a potential specific therapy that may aid early lung repair. However, its clinical usefulness is still limited by its rapid degradation and difficulties in achieving adequate delivery to the distal lung without unacceptable systemic effects [41], [42]. Thus it is important to find a more sustained delivery of KGF without inducing an immune response.

To enhance the level and consistency of expression of KGF in the distal lung we developed a new stem cell-based approach using an inducible Tet-on lentiviral vector (LV)-mediated KGF gene delivery system [43]. Bone marrow stem cells can be efficiently transduced by lentivirus, and maintain their self-renewal and differentiation abilities, so we hypothesized that lentivirally transduced bone marrow stem cells could serve as a constant but controlled pool of KGF within the lung parenchyma.

We demonstrate here, for the first time, that this combined cell/gene therapy is not only able to deliver KGF to the injured lung parenchyma, but that this KGF attenuates bleomycin-induced lung injury. We report that bone marrow derived cells expressing KGF in the lung are able to reduce the histological hallmarks of bleomycin-induced fibrosis, as well as induce proliferation of alveolar type II cells and decrease collagen levels within the lungs. Finally, comparing two different strategies using MSCs and HSCs as therapeutic vectors, our data suggests that progeny of HSCs may be better vehicles for gene therapy to ameliorate lung fibrosis.

## Materials and Methods

### Lentiviral Vector Constructs

The Tet-On inducible lentiviral vector was based on the pRRL-cPPT-hPGK-mcs-WPRE with the reporter gene MuSEAP encoded under the control of the hPGK promoter (kindly provided by Dr. Olivier Danos, UCL, London) [43]. To introduce the sequence of IRES-eGFP or mKGF-IRES-eGFP, the MuSEAP was excised using MluI/EcoRV enzymes. The IRES-eGFP sequence was amplified from an intermediate vector, and restriction sites were introduced by PCR (forward 5′ acgcacgcgtgcccctctccctccc 3′; reverse 5′ acgcgatatctcgagtgcggccgcttta 3′), and then inserted into MluI/EcoRV sites. Using the same strategy, the mKGF-IRES-eGFP sequence was amplified and restriction sites introduced by PCR (forward 5′ acgcacgcgtatgcacaaatggatactgac 3′; reverse 5′ acgcgatatctcgagtgcggccgcttta 3′), and inserted into MluI/EcoRV sites. The mKGF cDNA was kindly provided by Dr. JA Whitsett (Cincinnati Children's Hospital Medical Center, Cincinnati, USA).

### Ethics Statement, Animals and Cells Lines

All animal experiments were performed in compliance with Home Office and institutional guidelines. C57Bl/6 CD45.1 mice were bred in-house and CD45.2 were purchased from Charles River (UK) and quarantined for at least 7 days prior to use.

293T and HeLa cells were obtained from the ATCC and were grown in RPMI or DMEM, supplemented with 10% heat-inactivated fetal calf serum (FCS) and 100 IU/ml penicillin and 100 µg/ml streptomycin (Gibco) at 37°C in a humidified atmosphere containing 5% CO_2_.

### Production and Titration of Lentiviral Vectors

Lentiviral vectors pseudotyped with the vesicular stomatitis G protein (VSV-G) were generated by transfection into 293T cells of a packaging construct, pCMVAR8.74; a plasmid producing the VSV-G envelope (pMD.G); and the vector itself as previously described [44]. Culture medium was collected at 48 h and 72 h, 0.45 µm filtered, concentrated approximately 400 fold by ultra-centrifugation (Beckman), aliquoted and stored at −80°C until used.

Viral titres of infectious particles were determined by overnight transduction of 5×10^4^ Hela cells with serial dilutions of the virus in a 12-well plate with 4 µg/ml Polybrene (Sigma-Aldrich, # 52495). After transduction, medium was changed to new RPMI medium containing 10 µg/ml doxycycline (Sigma Aldrich) supplemented with 10% Tet-system-proved FCS (BD Biosciences, Clontech). Seventy-two hours later cells were analyzed for eGFP expression by flow cytometry.

### Isolation, Culture, and Lentiviral Gene Transfer into Murine MSCs

A frozen vial of murine MSCs (from male C57Bl/6 mice: courtesy of Tulane Centre of Gene Therapy, New Orleans, USA) was thawed and expanded by two passages as previously described [15]. After expansion, MSCs were transduced with lentiviral vectors carrying the eGFP reporter gene or KGF-eGFP. For transduction, cells were seeded at 2000 cells/cm^2^ in a T-75 cm^2^ flask. The following day virus particles were added at a multiplicity of infection (m.o.i.) of 50 for 16 hours. Cells were washed and fresh medium added with Doxycycline (10 µg/ml) for 5 days to induce transgene expression. To enrich eGFP or KGF-eGFP expressing cells, MSCs were sorted using the flow-activated sorter Mo-Flo (Becton-Dickinson, Oxford, UK). The eGFP positive fraction was cultured for 7 or 10 days. MSCs used were between passages 10–12.

### *In Vitro* Culture Differentiation Assay

Differentiation assays were performed as described [14]. Transduced MSCs were plated at a density of 2500 cells/cm^2^ under the same medium conditions as above for 24 to 48 hours. After attachment, cells were washed with basic differentiation medium, consisting of DMEM/2% FBS with penicillin/streptomycin, and then replaced with basic differentiation medium containing specific differentiation supplements. Osteogenic and adipogenic supplements were used as described [16]. Differentiation medium was changed every 2–3 days and cultured for 14 days.

### Gene Transfer into Murine Hematopoietic Stem Cells

Bone marrow from 8-12-week-old C57Bl/6 CD45.1 mice was obtained by flushing the femurs, tibias and iliac crests. Lineage negative cells were purified using the Murine Hematopoietic Progenitor Enrichment Cocktail (Stem Cells Technologies, Vancouver, Canada) according to the manufacturer's instructions. Lineage negative cells (Lin-) were pre-stimulated by culture (at 0.6-1×10^6^ cells/ml) in StemSpan (Stem Cell Technologies) serum-free medium containing murine SCF, Flt-3 and TPO at 50 ng/ml, and IL-6 at 10 ng/ml (all from Peprotech, London, UK), supplemented with 2% FCS, 100 IU/ml penicillin and 100 µg/ml streptomycin, at 37°C, 5% CO_2_ for 6 hours. Viral supernatants were added at m.o.i 30 (based on FACS titre) with 4 µg/ml Polybrene for 12 hours. Cells were washed and injected or re-seeded to analyse transduction efficiency or methylcellulose assay.

### Hematopoietic Progenitor Assays

Lin- cells were seeded in murine MethoCultGF M3434 medium containing SCF, IL-3, IL-6, and EPO (Stem Cell Technologies) and doxycycline 10 µg/ml to induce gene expression, at 10^4^ cells/ml/35 mm^2^ petri dish (in triplicates). After 7 days 0.25 ml of methylcellulose without cytokines (MethoCultGF M3234), but with or without doxycycline was added to the dishes to maintain gene expression induction. Both GFP+ and GFP- colonies were scored after 12 days by fluorescence microscopy under an inverted Leica DMIL microscope with a UV lamp (Leica Microsystems, Heerbrugg, Switzerland).

### Bone Marrow Repopulation and Peripheral Blood Chimerism Analysis

A total of 17 eight-week-old male C57Bl/6 CD45.2 recipient mice were lethally irradiated at 1200 cGy. 0.6×10^6^ mouse Lin- cells from donor C57Bl/6 CD45.1 mice were injected intravenously into the tail vein in 100 µl of PBS. 7 weeks after bone marrow transplantation, mice received doxycycline in drinking water (2 mg/ml) with 3% sucrose (Fisher laboratories). One week after gene induction, 100–200 µl of peripheral blood was collected in heparinized tubes, and analyzed for both chimerism and eGFP expression by flow cytometry. Red blood cells were lysed using ammonium chloride (Stem Cell Technologies, #07850). For chimerism and multilineage analysis, cells were stained with rat anti-mouse CD45.1-PE, CD45.2-APC and CD3-PE-Cy5 antibodies or corresponding isotype controls (for donor CD45.1 cells and recipient CD45.2 cells, lymphocytes and eGFP). Alternatively cells were stained with rat anti-mouse CD45.1-APC, Gr1-PE and Mac1-PE-Cy5 antibodies (for donor CD45.1 cells, Granulocytes, Macrophages and eGFP cells). All antibodies were purchased from BD Biosciences, Oxford, UK. Stained cells were analyzed by flow cytometry LSR-II (Beckton-Dickinson).

### Bleomycin-Induced Lung Injury and MSC or Lin- HSC Administration

For the MSC studies, a total of 23 eight-week-old male C57Bl/6 mice were exposed to bleomycin (2 mg/kg, KyowaHakk, UK) or saline by oropharyngeal (o.p.) instillation (50 µl/mouse) under light isofluorane-induced anaesthesia. Non-transduced or transduced MSCs with LV-eGFP or LV-KGF-IRES-eGFP (0.5×10^6^ in 100 µl of PBS) were injected intravenously by tail vein 8 hours after bleomycin instillation. Three days later, a second dose of 0.5×10^6^ of MSCs was injected. Mice were maintained on drinking water with doxycycline (2 mg/ml) and 3% sucrose to induce the gene expression during the whole experiment. Mice were weighed on the day of bleomycin administration and every 2 days until the end of the experiment. Animals were sacrificed 14 days after bleomycin administration, and the left lungs removed and used for RNA isolation. The remaining lung was inflated with 4% PFA in PBS at a pressure of 20 cm H_2_O, followed by removal of the inflated lungs *en bloc* and immersion in fresh fixative. Lungs were then processed for immunohistochemistry or, in some cases for flow cytometry analysis (see below).

For the HSCs studies, 15 mice underwent bone marrow transplantation with transduced Lin- cells and received bleomycin (2 mg/kg) or saline. Nine weeks post transplantation, 3 mice were given o.p. saline and doxycycline (Saline + KGF), 6 mice o.p. bleomycin without doxycycline (BLM), and 6 mice o.p. bleomycin with doxycycline (BLM+KGF). When used, doxycycline was maintained in the drinking water (2 mg/ml) with 3% sucrose until the end of the experiment. Mice were weighed on the day of bleomycin administration and every 2 days until the end of the experiment. Mice were sacrificed 14 days after bleomycin administration. Two extra mice underwent bone marrow transplantation with untransduced Lin^−^ cells, and received o.p. saline with doxycycline. The data from these 2 mice were used only for the type II alveolar cell proliferation analysis.

### Tissue Processing and Immunohistochemistry

Inflated lungs were fixed in 4% PFA for 4 hours at 4°C and then incubated overnight in 15% sucrose in PBS at 4°C. Tissue was then dehydrated in 70% ethanol and embedded in paraffin. Sections (4 µm) were stained with Haematoxylin and Eosin or Martius Scarlet Blue stain. For semi-quantitative analysis of fibrosis by Ashcroft Score, four different levels separated by 100 µm were analysed by two blinded independent investigators [2].

For all antibodies used, sections required microwave antigen retrieval (10 mM sodium citrate, pH 6 for 10 minutes), and were quenched for endogenous peroxidase with 1.6% H_2_O_2_. The sections were immunostained with anti-eGFP antibody (Rabbit polyclonal: Molecular Probes, Invitrogen, UK: 1∶500), anti-Ki67 (Rat monoclonal: Dako, UK: 1∶25), anti-Surfactant Protein C (Rabbit polyclonal: Chemicon, 1∶100), anti-FGF-7 (KGF) (Rabbit polyclonal: Santa Cruz, 1∶100) and anti-Alpha Smooth Muscle Actin (α-SMA) (Mouse monoclonal: Sigma, 1∶1000). For GFP and α-SMA stainings, biotinylated secondary antibody (1∶250) was used and immunoreactivity was detected using the ABC peroxidase-based system in combination with 3, 3′ diaminobenzidine, following the manufacturer's protocol. For KGF staining, the REAL™ Envision™ Dual Detection System (Dako) was used in combination with 3, 3′ diaminobenzidine, following the manufacturer's protocol. For Ki-67 and SP-C co-staining, a secondary goat anti-rat-Alexa 555 was used for Ki-67, and goat anti-mouse-Alexa 488 was used for SP-C (Molecular Probes, Invitrogen, UK). Absent and/or non-specific primary negative controls were included. For secondary antibodies conjugated with fluorochromes, sections were incubated with Sudan Black (0.1% in 70% ethanol) for 30 mins to reduce autofluorescence.

### Determination of Total Lung Collagen

The total collagen content of the left lung was determined by measuring hydroxyproline content in aliquots of pulverized left lung as described previously [45]. Hydroxyproline was quantified by reverse-phase high performance liquid chromatography (HPLC) of 7-chloro-4-nitrobenzo-oxao-1,3,-diazole-derived acid hydrolysates; the total left lung collagen was then calculated in milligrams, assuming that lung collagen contains 12.2% (w/w) hydroxyproline.

### Whole Lung Single-Cell Suspensions for Flow Cytometry

A single-cell suspension was obtained from murine lungs as described [46] with minor modifications. Saline-perfused lung samples were finely minced with razor blades and enzymatically digested for 1 h at 37°C in a solution of 0.1% of Collagenase A (Roche, # 103578), 2.4 U/ml Dispase II (Roche, # 295825) with 2.5 mM CaCl_2_ and DNase 0.4 mg/ml (Sigma-Aldrich, # D4513). The resulting digestion was then filtered through a 70 µm cell strainer (BD Biosciences, # 352340). Cells were washed with HBSS+ solution two times before staining with the rat anti-mouse CD45.1-PE and CD45.2-APC (BD Biosciences, Oxford, UK) for 30 minutes at 4°C. After staining, all single cell suspensions were washed with PBS 2% FCS and stained with DAPI (100 ng/ml) for exclusion of dead cells. Analysis was performed using an LSR-II flow cytometer (Becton-Dickinson).

### Quantitative Real-Time RT-PCR

Total RNA from frozen powdered lung tissue was isolated with TRIzol reagent as per the manufacturer's protocol. RNA was DNase-treated using a DNAfree kit (Ambion, UK). Random hexamers were used as the primer for reverse transcription (RT) of 1 µg of total RNA using the Superscript VILO™ cDNA synthesis kit (Invitrogen, UK) following the manufacturer's instructions. Real time RT-PCR was conducted using the Platinum SYBR Green qPCR SuperMix UDG (Invitrogen, UK) on a LightCycler 1.5 Real-time Detection System (Roche, UK) and analysed using LightCycler Real-time PCR Detection System Software Version 3.5. Cycling conditions were as follows: one cycle of 50°C (2 mins), 95°C (2 mins); 45 cycles of 95°C (5 secs), 55°C (5 secs), 72°C (15 secs). For each gene, crossing point (Cp) values were determined and normalized by subtraction of the Cp value for HPRT (generating a ΔCp value). Relative change was determined by subtraction of the ΔCp value for the control sample from the ΔCp value for the treated sample (ΔΔCp value). Fold change was subsequently calculated using the formula 2^−ΔΔCp^. All murine primers used were: HPRT (Forward 5′-TCATTATGCCGAGGATTTGG-3′; Reverse 5′-ACAGAGGGCCACAATGTGAT-3′), Col1a1 (Forward 5′-TCATGGCTTCTCTGGTCTC-3′; Reverse 5′-CCGTTGAGTCCGTCTTTGC-3′), KGF (Forward 5′-TTGACAAACGAGGCAAAGTG-3′; Reverse 5′- CCCTTTGATTGCCACAATTC-3′). TNFα (Forward 5′-CAAATGGCCTCCCTCTCAT-3′; Reverse 5′-CACTTGGTGGTTTGCTACGA-3′), CCL2 (Forward 5′-AGCTCTCTCTTCCTCCACCAC-3′; Reverse 5′-CGTTAACTGCATCTGGCTGA-3′) and CCL9 (Forward 5′-TACTGCCCTCTCCTTCCTCA-3′; Reverse 5′-AATTTCAAGCCCTTGCTGTG-3′).

### KGF Quantification by ELISA

Quantification of KGF protein by ELISA of MSC supernatants, plasma derived from peripheral blood, or lung powder from the HSC experiment, was performed according to the manufacturer's instructions (R&D systems).

### Statistical Analysis

Data were assessed for their suitability to be analyzed using parametric or non-parametric statistical tests and then underwent Student's t-test, One-Way ANOVA, Two-Way ANOVA (with Student-Newman-Keuls *post hoc* analysis) or a Two-Way Repeated Measures ANOVA with one factor repetition (for analysis of mouse bodyweight changes). For Ashcroft score analysis of lung fibrosis the non-parametric Mann-Whitney test was used. P values<0.05 were considered significant.

## Results

### Characterization of MSCs and Expression of KGF after Lentiviral transduction

**Figure 1:**
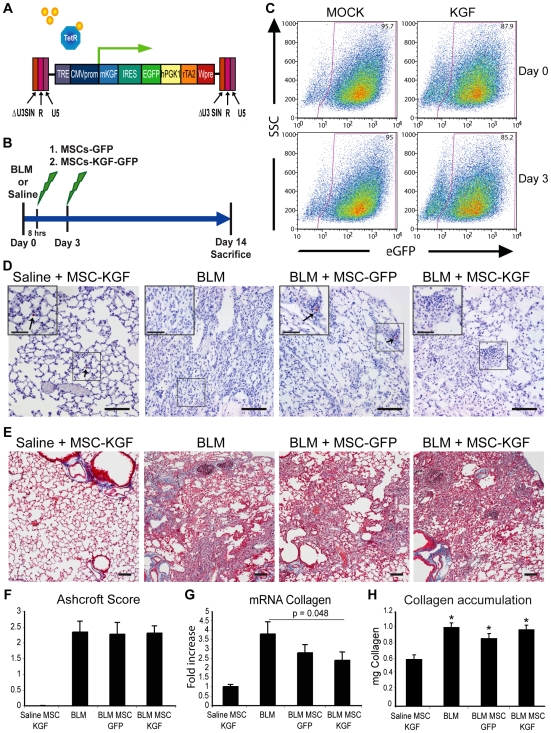
*In vitro* characterization of MSCs expressing KGF and their therapeutic potential in bleomycin-induced lung injury. A) Schematic representation of the lentiviral vector used in this study. B) Schematic of experimental design. C57Bl/6 mice received bleomycin (BLM) by o.p. instillation, followed 8 h later by i.v. injection of 0.5×10^6^ MSCs-eGFP or MSCs-KGF-eGFP, repeated three days later and then sacrificed at day 14. C) eGFP expression of transduced MSCs at the day of injection. D) Rare MSCs-eGFP and MSC-KGF-eGFP cells are detected in the lung after injury by immunohistochemistry (brown stained cells, arrows). (Scale bar = 100 µm. Inserts, scale bar = 50 µm). E) Representative images of Martius Scarlet Blue stained lung sections. (Scale bar = 100 µm). F) Comparison of morphometric analysis using Ashcroft Score, 14 days after bleomycin, showed no reduction of fibrosis with transduced MSCs. Bars represent means ± SEM. G) RT-PCR analysis for Collagen 1α1 mRNA from lung extracts showed significant differences between bleomycin and bleomycin +MSCs-KGF-eGFP groups while H) there was no difference in lung collagen accumulation as measured by reverse-phase HPLC quantification of lung hydroxyproline. Statistical differences were detected between saline and bleomycin groups regardless of MSC administration (*p<0.05). Bars represent means ± SEM. Statistically significant differences are indicated by p-values from a One-Way ANOVA test.

We first examined delivery of KGF using MSCs. Murine MSCs were cultured as previously described [15]. After expansion by two passages, the MSCs were transduced with KGF-eGFP (MSC-KGF) or an eGFP control (MSC-GFP) using an inducible lentiviral vector (to be able to compare with parallel studies in HSCs) (Fig. 1A). *In vitro* experiments confirmed that the gene expression was induced only in the presence of doxycycline as measured by GFP expression using flow cytometry (not shown). One week after transduction, MSCs expressing GFP were purified by cell sorting and expanded in the presence of doxycycline. The multipotent differentiation capacity of the transduced MSCs was confirmed by their differentiation into adipocytes and osteoblasts as shown by Oil Red O (adipocyte) and Alizarin Red S (osteoblast) staining of *in vitro* differentiation cultures (Figure S1A).

### Murine MSCs with or without KGF Decrease Collagen 1 Production but Do Not Attenuate the Histological Damage in Bleomycin Induced Fibrosis

Previous studies have shown that early but not late delivery of MSCs can attenuate the inflammatory response to bleomycin induced lung damage and ameliorate the fibrotic effects. We used MSCs to deliver KGF in an attempt to further reduce the damaging effects of bleomycin and subsequent fibrosis. Mice were treated with o.p. bleomycin or saline (Control) followed by the delivery of PBS (Control) or 1×10^6^ transduced MSCs separated in two doses: first dose by intra-venous injection of 5×10^5^ MSC-KGF-GFP or MSC-GFP or 100 µl PBS at 8 hours after bleomycin (day 0), and a second dose on day 3 after damage (Fig. 1B). In order to induce the KGF expression, we added doxycycline to the drinking water from day 0 after bleomycin administration. Flow cytometry analysis of a portion of the MSCs harvested for injection showed doxycyline induced GFP expression in over 85% of both MSC-GFP and MSC-KGF-GFP (Fig. 1C). Furthermore, we measured the KGF expression in the supernatants of the cultured cells by ELISA, and confirmed that injected MSC-KGF-GFP cells but not MSC-GFP cells were secreting KGF (Figure S1B). As bleomycin injury causes a decrease in mouse bodyweight, we analyzed the weight of the mice over the 14 days following damage. Although a slightly lower bodyweight loss was observed in mice treated with MSCs, we found no significant differences between the bleomycin treated groups (Figure S1C). In order to localize the transduced MSCs within the lungs, anti-GFP immunohistochemistry was performed in lung sections 14 days after bleomycin administration. GFP staining demonstrated very low numbers of cells expressing GFP in both saline and bleomycin treated mice (Fig. 1D, inserts). These data were confirmed by flow cytometry of the digested left lung lobe, revealing approximately 0.1% of total lung cells were GFP positive (Figure S1D). Consistent with the low levels of MSC engraftment at 14 days, no significant difference in the KGF mRNA level among the bleomycin groups was detected (Figure S1E). Systemic KGF levels were also assessed by measuring KGF in plasma from peripheral blood samples. No KGF protein was detectable by ELISA in any of the samples tested (data not shown); these data suggest that little or no KGF is reaching the systemic circulation.

Histological examination using H&E and Martius Scarlet Blue Staining of lung architecture and collagen deposition revealed typical features of bleomycin damage in all three treated groups (Fig. 1D and E). Modified Ashcroft scores were performed, in a blinded fashion, to quantify lung damage. This analysis revealed no differences between the bleomycin groups demonstrating that neither MSC-GFP nor MSC-KGF-GFP improved the lung architecture (Fig. 1F). To determine if the MSCs were able to reduce the characteristic collagen deposition in the lungs after bleomycin-induced damage in mice, we performed real time PCR for Collagen-1α1 mRNA and a biochemical quantification of the collagen protein content by HPLC. Surprisingly, we found that both MSC-GFP and MSC-KGF-GFP reduced collagen mRNA levels but only MSC-KGF-GFP treated mice showed a statistically significant decrease compare to the bleomycin only treated group (Fig. 1G). In contrast, HPLC determination of hydroxyproline content, as a surrogate marker for collagen protein levels, demonstrated no significant differences between the bleomycin groups – in agreement with the Ashcroft scores. Bleomycin treatment per se resulted in significantly increased levels of left lung collagen, irrespective of MSC treatment (Fig. 1H). Taken together, in our experiments MSCs do not reduce the accumulation of lung collagen or the severity of fibrosis, at least at 14 days post bleomycin. However, given the apparent reduction in collagen-1α1 mRNA levels at 14 days, we cannot rule out that ongoing fibrosis may be attenuated at later time points.

Hence we have efficiently induced both *in vitro* and *in vivo* GFP and KGF expression in transduced MSCs. However the delivery of MSCs, injecting twice at early stages after bleomycin exposure (days 0 and 3), did not reduce the fibrosis assessed biochemically and histologically.

### Characterization of Lentiviral Ttransduction of KGF-eGFP into Lineage Negative Hematopoietic Stem Cells

**Figure 2:**
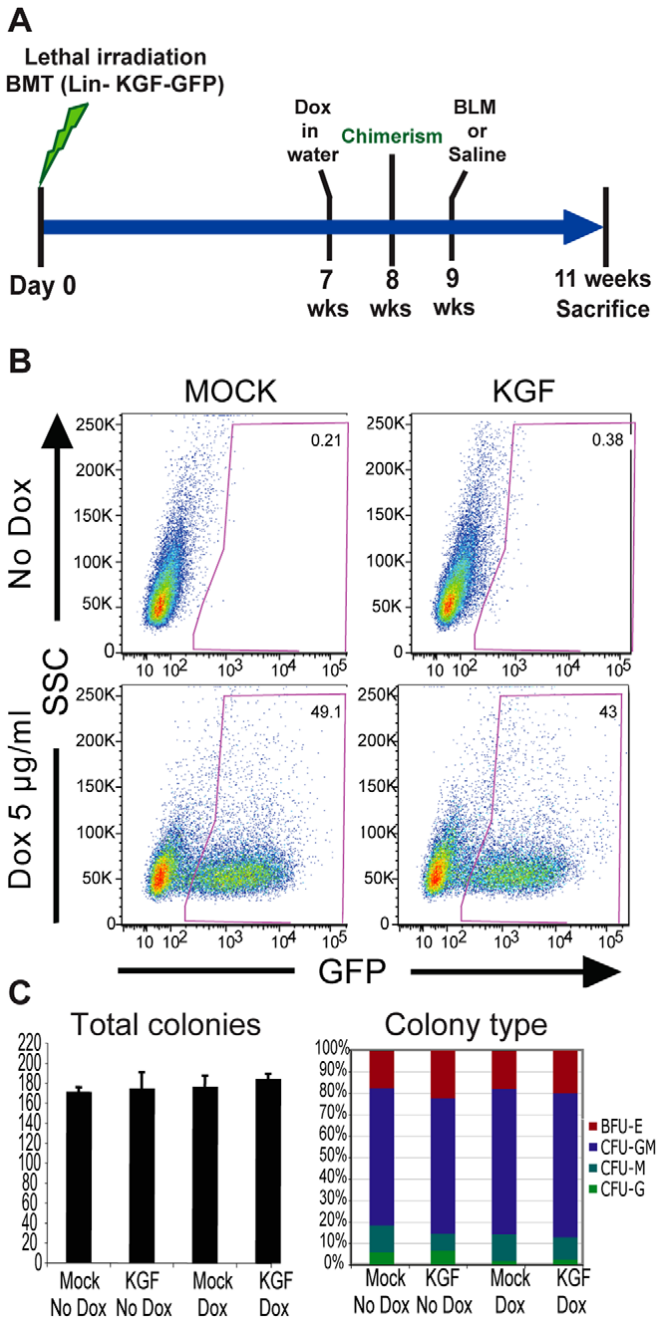
Lineage negative HSCs KGF-eGFP transduction and differentiation potential. A) Schematic of experimental design. 8 weeks-old C57Bl/6 mice were lethally irradiated followed by intravenous injection of 0.6×10^6^ transduced lin- HSCs (BMT). 7 weeks after BMT, mice received doxycycline in drinking water to induce gene expression and chimerism was analysed one week later. 9 weeks after BMT, mice received bleomycin by o.p. instillation and were sacrificed at day 14. B) Flow cytometry analysis showed eGFP expression *in vitro* after adding doxycycline to lin- HSCs transduced with LV-eGFP (MOCK) and LV-KGF-eGFP. C) Amount and type of colonies in methylcellulose after culture of transduced Lin^-^ HSCs with MOCK or KGF-eGFP was unchanged. BFU-E, erytrocyte; CFU-G, granulocyte; CFU-M, myeloid; CFU-GM, mixed granulocyte and myeloid.

In parallel to the experiments using MSCs, and in order to address the therapeutic potential of other bone marrow cell populations, we explored the possibility of using bone marrow transduced HSCs as a cell therapy vehicle. In these experiments we harvested and transduced lineage negative (Lin-) hematopoietic stem cells with KGF-expressing lentivirus (HSC-KGF) or the eGFP control (HSC-GFP; Mock) and then performed bone marrow transplants (BMT). Seven weeks after BMT we administered doxycycline in drinking water to induce KGF and GFP expression, followed by bleomycin damage at week 9 after BMT (Fig. 2A). HSC-KGF transplanted mice received o.p. saline (n = 3) or bleomycin (n = 6); a third group of mice received bleomycin only (n = 6).

*In vitro* treatment of the Lin- HSCs with doxycycline revealed GFP expression rates between 40-50% with both lentiviruses, compared with less than 0.4% in no doxycycline controls, demonstrating good transduction rates and an efficient and tightly controlled gene induction by doxycycline (Fig. 2B). Importantly, KGF expression did not affect the colony formation and differentiation potential of HSCs compared to Mock in methylcellulose assays (Fig. 2C), confirming that KGF did not alter the potential of these cells to differentiate into the mature blood lineages *in vitro*.

Eight weeks post-BMT, we analyzed blood chimerism by flow cytometry using CD45.1 (donor cells) and CD45.2 (recipient cells) markers. The average degree of chimerism in these mice was 53%, and after one week of doxycycline treatment approximately 10% of all the donor circulating cells were expressing GFP (Table 1 and Figure S2A). Recipient mice showed donor bone marrow multi-lineage engraftment and, in mice treated with doxycycline, an efficient transduction in each lineage (granulocytes, macrophages and lymphocytes). The GFP levels within these cell populations were similar to the GFP levels from the total donor blood cells, as measured by flow cytometry (Table 1 and Figure S2B).

**Table 1:** Multilineage engraftment 8 weeks after bone marrow transplantation with transduced Lineage negative HSCs.

Chimerism	GFP donor cells	GFP Granulocytes from Donor	GFP Macrophages from donor	GFP Lymphoid from Donor
53.32±10.33	9.73±5.12	17.01±7.79	16.98±7.75	7.82±4.29

Expressed in percentages and standard deviations.

* All mice were exposed to doxycycline in drinking water for a week pre analysis (n = 15).

### Transplantation of Lineage Negative Cells Expressing KGF Protects from Bleomycin Induced Fibrosis

**Figure 3:**
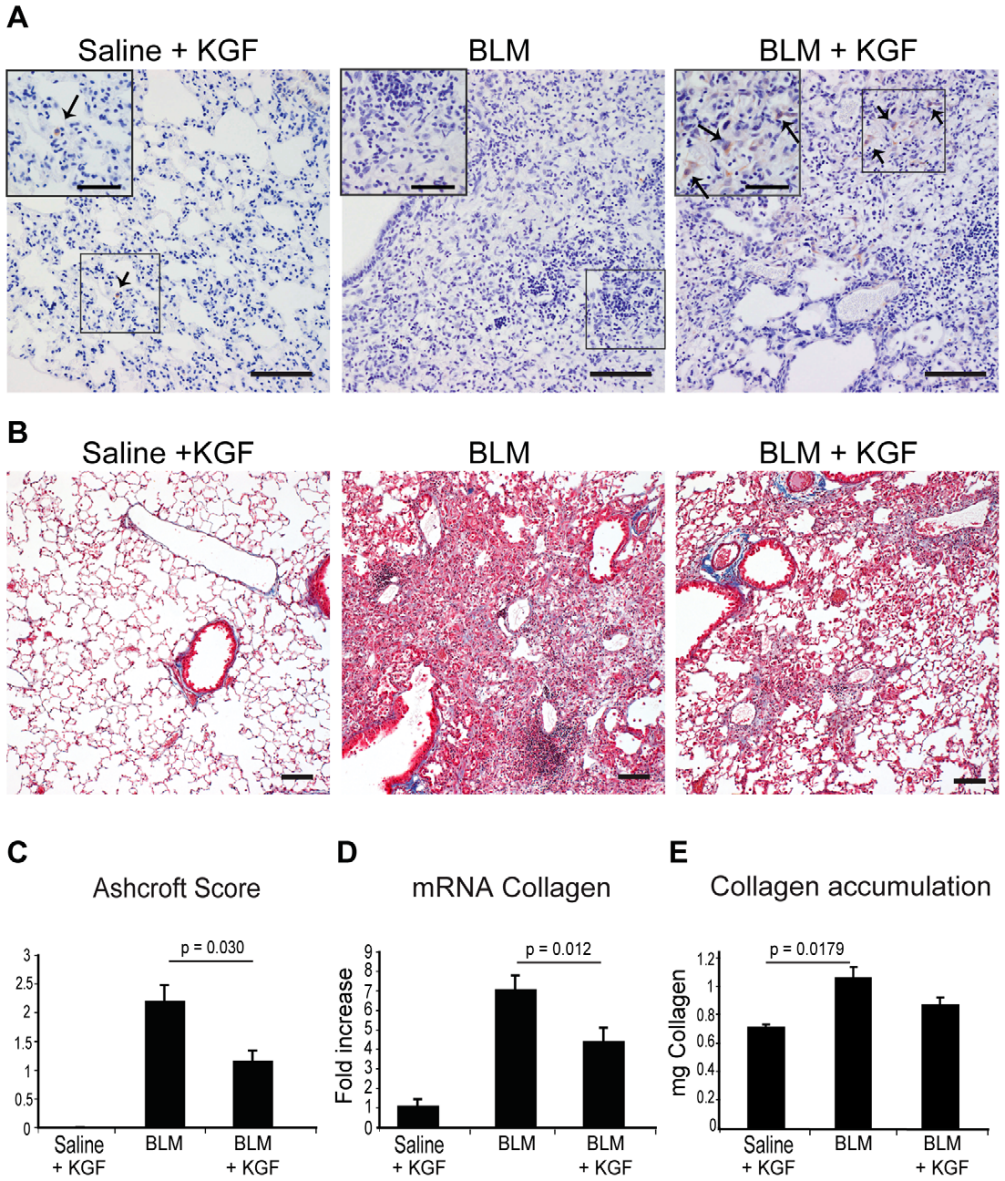
Therapeutic potential of KGF expressing Lin- HSCs in bleomycin-induced lung fibrosis in mice. A) GFP immuno-staining 14 days after bleomycin demonstrated brown stained cells (arrows) in mice given doxycycline in drinking water. (Scale bars = 100 µm; inserts, scale bars = 50 µm). B) Martius Scarlet Blue stained lung sections showed a reduction of fibrosis in mice expressing KGF-eGFP. Scale bar = 100 µm. C) Semi-quantitative representative morphometric analysis using Ashcroft Score, confirmed the reduction in lung fibrosis when KGF is expressed by bone marrow cells. (p = 0.03; Mann-Whitney). D) RT-PCR analysis for Collagen 1α1 mRNA from lung extracts showed reduced collagen in the bleomycin + KGF-eGFP group *versus* bleomycin only group (p = 0.012; One-Way ANOVA). E) KGF attenuated lung collagen accumulation after bleomycin instillation in mice, as measured by reverse-phase HPLC quantification. A statistical significant increase was detected in saline *versus* bleomycin only group, but not the HSC-KGF treated group. (p = 0.0179, One-Way ANOVA). All bars represent means ± SEM.

Nine weeks post-BMT mice were treated with o.p. bleomycin (2 mg/kg) or saline (Control), and sacrificed 14 days later. To localize bone marrow derived cells from the transduced HSCs, we analysed GFP expression within the damaged lungs. Immunohistochemical analysis of the lung showed BMDCs expressing GFP only in the HSC-KGF mice treated with doxycycline at a low percentage (Fig. 3A). This result was confirmed by flow cytometry, where approximately 2% of cells were GFP positive (Figure S3A and Table 2).

**Table 2:** Donor blood cells and donor GFP positive blood cells from single cell lung suspensions.

Group	Donor Blood Cells	GFP donor Blood cells	GFP non Blood cells	Recipient Blood cells	Total blood Cells/lung
BLM	13.05±4.47	0.18±0.10	0.08±0.007	12.14±5.15	25.19±2.16
BLM + KGF-eGFP	17.1±3.56	1.68±0.73	1.08±1.09	10.02±2.41	27.13±5.14

Numbers expressed in percentages and standard deviations. Freshly isolated lung cells were used for flow cytometry analysis. Similar percentage of total and donor blood cells was detected in the bleomycin groups.

Bleomycin injury alone caused a significant decrease in mouse bodyweight over time compared with saline-treated animals (effect of treatment/time, p<0.05), with individual timepoints from day 7 onwards being highly significantly different. The change in bodyweight for HSC-KGF transplanted bleomycin-treated animals was significantly different from both saline-treated and bleomycin only-treated animals, suggesting a partial amelioration in bodyweight loss with HSC-KGF transplantation (effect of treatment/time, p<0.05), although no individual time points were significantly different (Figure S3B).

Fourteen days after bleomycin administration, lungs were harvested and histologically assessed with H&E and Martius Scarlet Blue staining (Fig. 3A and B). Modified Ashcroft scoring, determined in a blinded fashion, showed that HSC-KGF treated mice had significantly lower Ashcroft scores than those receiving bleomycin alone (Fig. 3C). The reduction in fibrosis was not only demonstrated by histology, but also by significantly reduced Collagen 1α1 mRNA levels compared to the bleomycin alone (p<0.012; Fig. 3D). HPLC quantification of hydroxyproline content demonstrated a significant increase in total lung collagen in mice given bleomycin alone compared with saline-treated animals (p = 0.0179; Fig. 3E), but there was no significant difference between saline-treated animals and HSC-KGF transplanted mice given bleomycin. These data suggest that HSC-KGF transplantation can attenuate lung collagen accumulation in this model, and confirms the efficiency of using these bone marrow derived stem cells expressing KGF as a system to reduce fibrosis.

### Lineage Negative Cells Expressing KGF Induce Epithelial Proliferation

**Figure 4:**
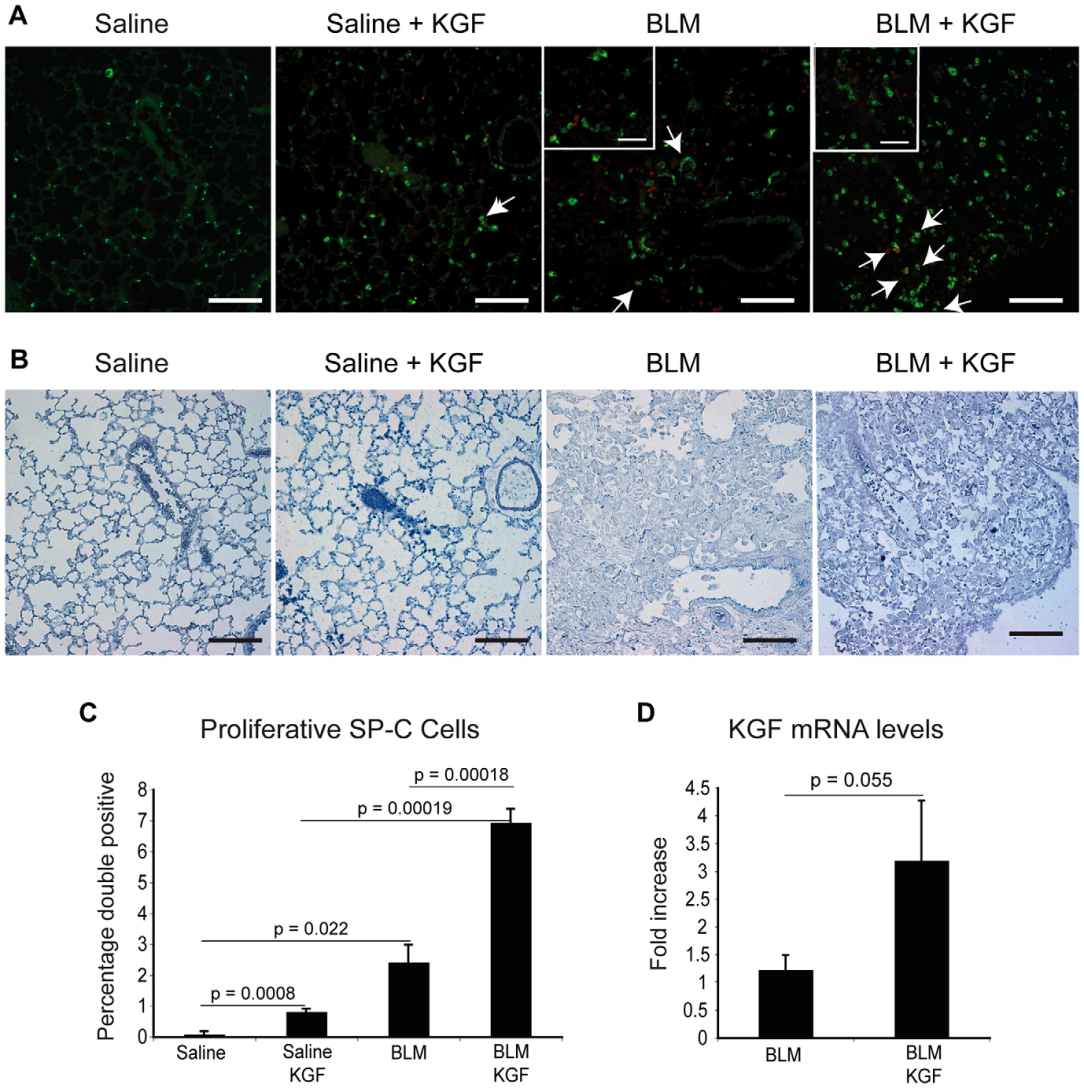
Bone marrow derived cells expressing KGF induced alveolar epithelial cell proliferation within the injured lung. A) Double immunofluorescence with lung epithelial type II marker Surfactant Protein C (SP-C) (green) and proliferation marker Ki-67 (red) on paraffin lung sections, 14 days after bleomycin administration. Bone marrow cells expressing KGF increased proliferation of alveolar type II cells in both saline and BLM groups. Arrows indicate proliferating lung epithelial cells. (Scale bars = 100 µm; inserts, scale bars = 50 µm). B) Contiguous light microscopy photomicrographs corresponding to A. C) Quantification of double positive SP-C and Ki-67 stained cells showed an increase in proliferating epithelial cells when KGF is expressed. Bars represent mean from 6-areas/lung sections counted ± SEM. (P-values from Two-Way ANOVA). D) qRT-PCR analysis for KGF mRNA from lung extracts showing increased levels of KGF in doxycycline treated mice. Bars represent means ± SEM. (P-values from Student's T-test).

KGF has been shown to induce type II pneumocyte proliferation both *in vitro* and *in vivo* [37], [47], [48]. Based on this potential, we were interested to determine whether the amelioration obtained from damage induced by bleomycin was associated with an increase in type II cell proliferation. To address this question, dual immunofluorescence for the proliferation marker Ki67 and the type II cell marker pro-SPC was performed and double positive cells counted from 6 independent areas, by histopathologists blinded to the mouse treatments (Fig. 4A). As lung tissue presents high levels of auto-fluorescence, tissues were treated with Sudan Black after the immunohistochemistry (See Materials and Methods). We detected a significant increase in double positive cells in bleomycin treated HSC-KGF mice compared to bleomycin controls (p = 0.0002) (Fig. 4C). Interestingly, in light photomicrographs of the analyzed areas, the proliferating epithelial cells were observed close to fibrotic lesions or regenerating areas surrounding lesions (Fig. 4B). Moreover, there was also a small but significant increase in double positive cells in HSC-KGF-saline animals compared to saline controls (p = 0.0008) suggesting some effect of KGF once activated by doxycycline in non-damaged epithelium and hence the potential importance of an inducible system. In order to correlate the increase of proliferation with KGF expression, we performed real time PCR for KGF. This analysis revealed an increase in KGF mRNA levels between HSC-KGF bleomycin treated mice compared to bleomycin treated controls, although this just failed to reach significance (Fig. 4D). To further investigate the presence and localisation of KGF in the fibrotic lung, we investigated KGF protein levels by immunohistochemistry and ELISA. We detected a low frequency of KGF-expressing cells in the lungs of mice from the saline group but, as expected, a higher frequency of KGF positive cells was detected in both bleomycin groups, particularly in fibrotic lesions and the surrounding area (Figure S4A, positive cells indicated with arrows). As KGF is a secreted protein, quantification of positive cells between the bleomycin groups was difficult to assess. However, in order to demonstrate that a sub-population of KGF positive cells originated from the transduced HSC-derived bone marrow cells, we performed immunohistochemistry for GFP on serial sections (dual staining was not possible, due to the incompatibility of staining protocols for KGF and GFP). Interestingly, we detected GFP positive cells that co-localised with KGF immunoreactivity, in both saline and bleomycin groups that were given doxycycline (Figure S4B, positive cells indicated with arrows).

We also measured the KGF protein levels in the lungs by ELISA of lung homogenates. KGF protein levels were reasonably high in the bleomycin group, and there was a trend for increased KGF protein levels in mice transplanted with HSC-KGF cells despite lower levels of fibrosis (Figure S4C). Interestingly KGF levels correlated with hydroxyproline content of the lungs after bleomycin injury in controls but not in HSC-KGF treated mice (which we would have predicted to be lower due to the reduced injury), suggesting that the raised levels were indeed due to the local KGF delivery by bone marrow derived cells (Figure S4D). These data are in agreement with the increased KGF mRNA levels detected by real time PCR, as well as the presence of GFP positive cells co-localising with KGF expression. Importantly, KGF was not detectable in peripheral blood ELISA suggesting a local rather than systemic therapeutic effect.

### Lineage Negative Cells Expressing KGF Reduce Pro-Inflammatory Cytokines, Chemokines and α-Smooth Muscle Actin Positive Myofibroblasts

**Figure 5:**
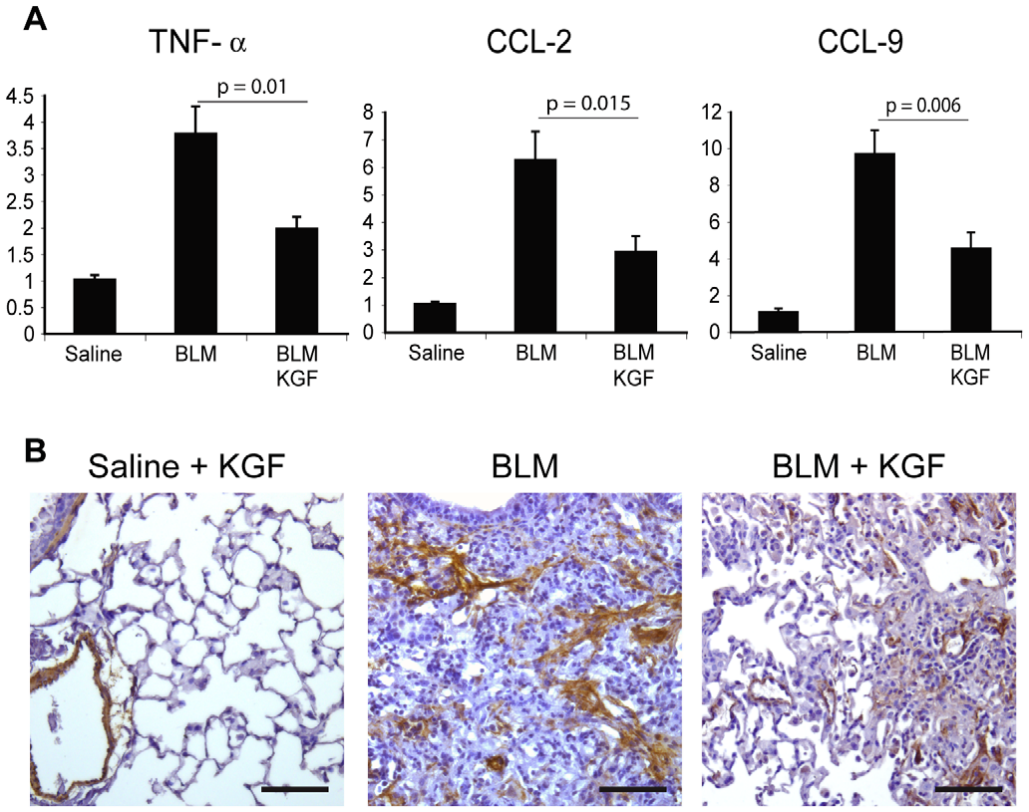
Bone marrow derived cells expressing KGF reduce inflammatory cytokines, macrophage chemo-attractants and α-sma. A) qRT-PCR analysis for inflammatory cytokines revealed significant reductions of TNF-α and CCL-2 and CCL-9 chemokine mRNA in lung extracts from doxycycline treated BLM-KGF mice compared with BLM treatment alone. Bars represent means ± SEM. (P-values from One-Way ANOVA). B) α-sma stained lung sections showed a reduction of myofibroblast-type cells in the lungs of mice treated with KGF-eGFP. Scale bar = 50 µm.

To assess effects on inflammation we examined levels of the pro-inflammatory cytokine TNF-α and the leukocyte chemoatractants CCL2, CCL9 by real time PCR. Bleomycin treated HSC-KGF mice had a very clear reduction of the mRNA levels of all three cytokines compared to bleomycin treated control mice (Fig. 5A), suggesting that KGF expression may reduce inflammation.

It is also well established that the central effector cell in the development of lung fibrosis is the myofibroblast. These cells are derived from the differentiation of fibroblasts, under the influence of pro-fibrotic factors such as TGF-beta. These cells are the main cell type responsible for the synthesis and deposition of extracellular matrix and the resultant structural remodelling that leads to the loss of alveolar function [49]. We performed immunohistochemical analysis to detect α –smooth muscle actin (α –sma) in the fibrotic areas of the lungs and detected a reduction in HSC-KGF treated mice compared with bleomycin alone (Fig. 5B). These data not only support the reduction of fibrosis observed and quantified by Ashcroft score (Fig. 3B and 3C), but also provide indirect evidence for the effect of KGF expression by HSCs on the reduction of factors, such as TGF-β1, responsible for mediating the pro-fibrotic effect.

## Discussion

The main purpose of this work was to demonstrate, as a proof of concept, that bone marrow transplantation using an inducible gene transfer system to deliver KGF could provide a successful therapy for lung injury in mice. We chose to deliver KGF due to its established role in pulmonary epithelial repair [37], [47], [48]. KGF induces type II cell proliferation in rabbits, rats and mice [38], [42], [48] with an associated increase in the protective surfactant proteins [38], [50]. Furthermore, KGF improves alveolar repair by enhancing cell spreading and motility [51].

As we believe that different bone marrow cell populations may have different therapeutic effects in the lung, we decided to study in parallel, MSCs and HSCs as vehicles to deliver KGF into the lung. The successful contribution of BMDCs into normal lung [11], [13], and after induced-lung injury [29], [31] encouraged us to attempt an HSCs-based gene therapy in the bleomycin-induced lung fibrosis model.

In order to eliminate the potential long-term side effects of KGF on HSC development, we opted for an inducible Tet-On lentiviral vector. We showed first that this system was working efficiently i.e. the transduced cells can be induced *in vitro* to express GFP and KGF and that the system was not leaky. The transduced cells repopulated the bone marrow of recipient mice and could be activated *in vivo* with doxycycline, as confirmed by GFP expression using flow cytometry. GFP (hence KGF) expressing cells were detected in approximately 10% of donor cells in the peripheral blood of these mice.

Based on previous reports, we expected that the delivery of KGF using MSCs in particular would be beneficial, as MSCs themselves have been also shown to have immuno-suppressive properties. Hence this therapy could be viewed as a combination of the MSC's immuno-suppression of the pro-inflammatory response and KGF mediated stimulation of epithelial proliferation and reduction of apoptosis [52]. An alternative strategy of reducing inflammation has successfully been used in the LPS induced acute lung injury model using MSCs to deliver Angiopoietin 1 [53]. Here MSCs reduced the inflammatory cell count in bronchial alveolar lavage (BAL) and also the level of pro-inflammatory cytokines. Interestingly in this study neither treatment with MSCs nor MSCs delivering Angiopoietin 1 significantly improved the histological score of the lung damage.

The failure of MSCs to alter the histopathological damage scores in our experiments is intriguing. MSCs can inhibit the activation of immune cells resulting in a reduction of inflammatory response. Mei et al noted that the delivery of MSCs resulted in near complete reduction of tissue cytokine expression in their LPS induced acute lung injury model [53] while others have noted reduced inflammation in the lung after MSC delivery in the bleomycin model [8], [17], [18]. It has been noted that MSCs must be delivered early in the bleomycin model to attenuate the resulting fibrosis [8], [18] correlating with the early inflammatory steps in the pathogenesis of bleomycin-induced lesions [54]. We delivered MSCs at the time of bleomycin injury and 3 days later. While the end points of our experiments have not allowed an investigation on early changes in inflammation the lack of an impact in the development of fibrotic lesions was clear although the reduction of Collagen 1α1 mRNA expression does suggest some sub-clinical effect. It has been shown that increased engraftment of the injected MSCs depend on tissue injury. The engraftment of endogenous eGFP-labelled MSC one week after endotoxin [30] and 3 weeks after elastase inhalation [9] was seen after the pulmonary insult but not in controls. However in our study and in some other recent studies very low engraftment levels in both undamaged and damaged lung at 14 days have been observed [18], [53], [55].

For the HSC-based gene therapy approach, we induced KGF/GFP expression before bleomycin administration based on previous studies in which they found that delivery of recombinant KGF early in the disease pathogenesis was essential for its amelioration [38]. In this study we show a significant reduction of the pathological hallmarks of bleomycin-induced lung fibrosis using HSCs-KGF, with a clear reduction in collagen 1α1 mRNA and collagen content, as well as in lung damage using a histological score analysis. Importantly, these results are reflected in the health state of the mice, with doxycycline treated mice showing reduction in weight loss. Moreover, we detected a significant increase in the mitogenic activity of alveolar type II cells in those mice with proliferating type II cells associated with fibrotic foci. We demonstrated the presence of KGF and GFP double positive cells in the lungs of mice treated with doxycycline demonstrating bone marrow derived cells within areas of damage expressing KGF. These data support the idea that the reduction in fibrosis observed may be due to, at least in part, the mitogenic capacity of KGF on type II alveolar cells. When we measured the KGF protein from the lungs extracts by ELISA, we detected an increase of KGF in the mice treated with HSC-KGF. However no protein was detected in the peripheral blood in any of the mice (data not shown), supporting the idea that the protective effect of KGF in the lungs is local rather than systemic. We also show a reduction in the expression of the pro-inflammatory cytokine TNF-α, and leukocyte chemoattractants CCL-2 and CCL-9 and finally, a reduction of α-sma in the fibrotic foci of the lungs from the mice treated with HSC-KGF, indicating a possible reduction of the pro-fibrotic effects of TGF-β1, a key factor in lung fibrosis.

Although several groups have used constitutive lentiviruses to tranduce HSCs for the treatment of inherited and acquired severe hematological or immunological disorders [56], very few groups have used this strategy for the treatment of lung diseases. Recently Zhang et al, have been able to treat pulmonary metastatic tumors in mice using lentiviral-vector transduced HSCs [57]. However, there are no reports in primary lung disease models of using bone marrow transplantation of lentivirus-transduced HSCs.

Of course, bone marrow transplantation is a rather drastic step for the treatment of pulmonary fibrosis in humans, and may not be the most practical strategy to be used clinically. However, we strongly believe that our results in this mouse model show that gene therapy for diffuse lung diseases is possible with hematopoietic stem cells, and that experiments are warranted to try and determine if a single group of differentiated cells is capable of similar results.

## Supporting Information

Figure S1 Characterization of MSCs and transduction analysis with eGFP and KGF-eGFP. A) Adipocyte (Oil red-O staining) and Osteogenic (Alizarin red staining) differentiation of transduced MSCs. (10x objective using inverted microscope). B) KGF protein levels were detected by ELISA from the supernatant of MSCs-KGF-eGFP but not from MSCs-eGFP on the day of injection into C57B/6 mice. C) Body weights post bleomycin treatment showed no significant differences in the weight loss induced by bleomycin. D) Flow cytometry to detect eGFP expressing MSCs from freshly isolated lung cells 14 days after bleomycin showed almost no eGFP cells in any group. E) qRT-PCR analysis for KGF mRNA from lung extracts shows a modest increase in KGF expression in MSCs-KGF-eGFP mice compared to control groups. Bars represent means +/− SEM.
(1.56 MB TIF)

Figure S2 Chimerism, transduction and multilineage in vivo engraftment analysis of HSCs. A) Flow cytometry of peripheral blood from recipient mice 7 weeks after BMT plus one week with doxycycline treatment. Donor KGF-eGFP expressing blood cells were detected within the donor bone marrow population labelled with CD45.1 in the presence of doxycycline. B) Multilineage engraftment analysis by flow cytometry. Blood markers for macrophages (Mac-1), granulocytes (Gr-1) and lymphocytes (CD-3) were used together with the donor marker (CD45.1). In each differentiated subpopulation, KGF-eGFP expressing cells were detected, demonstrating an efficient bone marrow repopulation and in vivo induction.
(7.89 MB TIF)

Figure S3 Expression of KGF-GFP in the lungs reduces body weight loss in mice after bleomycin administration. A) Flow cytometry of left lung digests detecting KGF-eGFP expressing cells 14 days after bleomycin administration. B) Body weights post bleomycin treatment showed doxycycline-induced expression of KGF from BMDC (BLM+KGF) reduces the weight loss induced by bleomycin.
(4.63 MB TIF)

Figure S4 Increased detection of KGF positive cells in mice expressing HSC-KGF correlates with GFP positive cells. A) KGF immuno-staining 14 days after bleomycin demonstrated frequent brown stained cells (arrows) in mice treated with doxycyline. (Scale bars = 50 um; inserts, scale bars = 25 um). B) GFP staining in serial lung sections showed a correlation of KGF and GFP expression in mice treated with doxycyline only. (Scale bar = 50 um, inserts, scale bars = 25 um). C) KGF ELISA from lung powder showed higher levels of KGF in mice treated with doxycyline. D) Correlation of KGF protein levels, measured by ELISA, with the collagen content of the lungs after bleomycin injury. Linear correlation values are indicated for each group.
(6.03 MB TIF)
