# Supplementary figures and images for: Bone Marrow Stem Cells Expressing Keratinocyte Growth Factor via an Inducible Lentivirus Protects against Bleomycin-Induced Pulmonary Fibrosis
Source: PLoS One. 2009 Nov 24;4(11):e8013. doi: 10.1371/journal.pone.0008013 (PMC2779453; doi:10.1371/journal.pone.0008013)

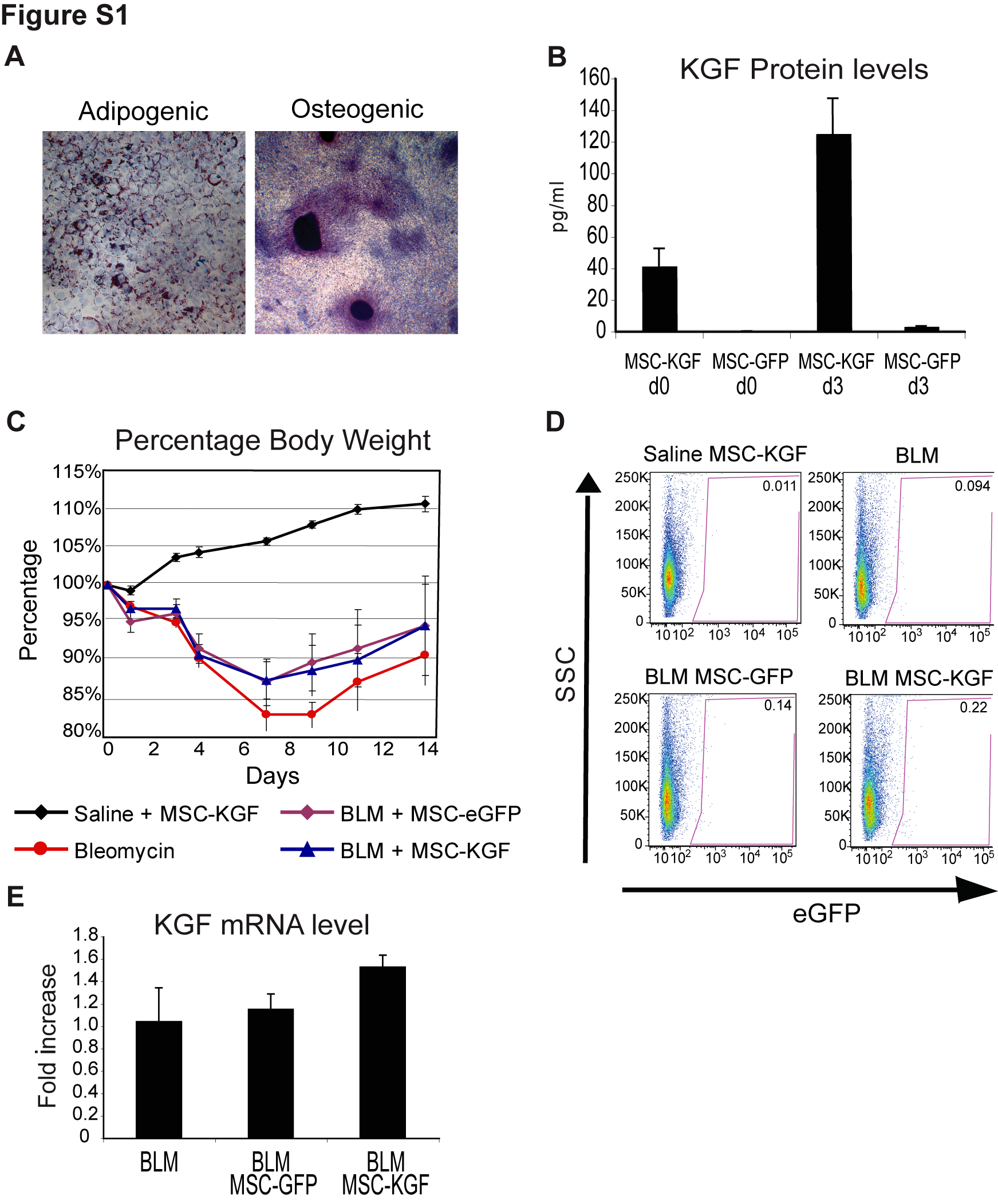

Supplement: Figure S1 — Characterization of MSCs and transduction analysis with eGFP and KGF-eGFP. A) Adipocyte (Oil red-O staining) and Osteogenic (Alizarin red staining) differentiation of transduced MSCs. (10x objective using inverted microscope). B) KGF protein levels were detected by ELISA from the supernatant of MSCs-KGF-eGFP but not from MSCs-eGFP on the day of injection into C57B/6 mice. C) Body weights post bleomycin treatment showed no significant differences in the weight loss induced by bleomycin. D) Flow cytometry to detect eGFP expressing MSCs from freshly isolated lung cells 14 days after bleomycin showed almost no eGFP cells in any group. E) qRT-PCR analysis for KGF mRNA from lung extracts shows a modest increase in KGF expression in MSCs-KGF-eGFP mice compared to control groups. Bars represent means +/− SEM. (1.56 MB TIF) [file pone.0008013.s001.tif]

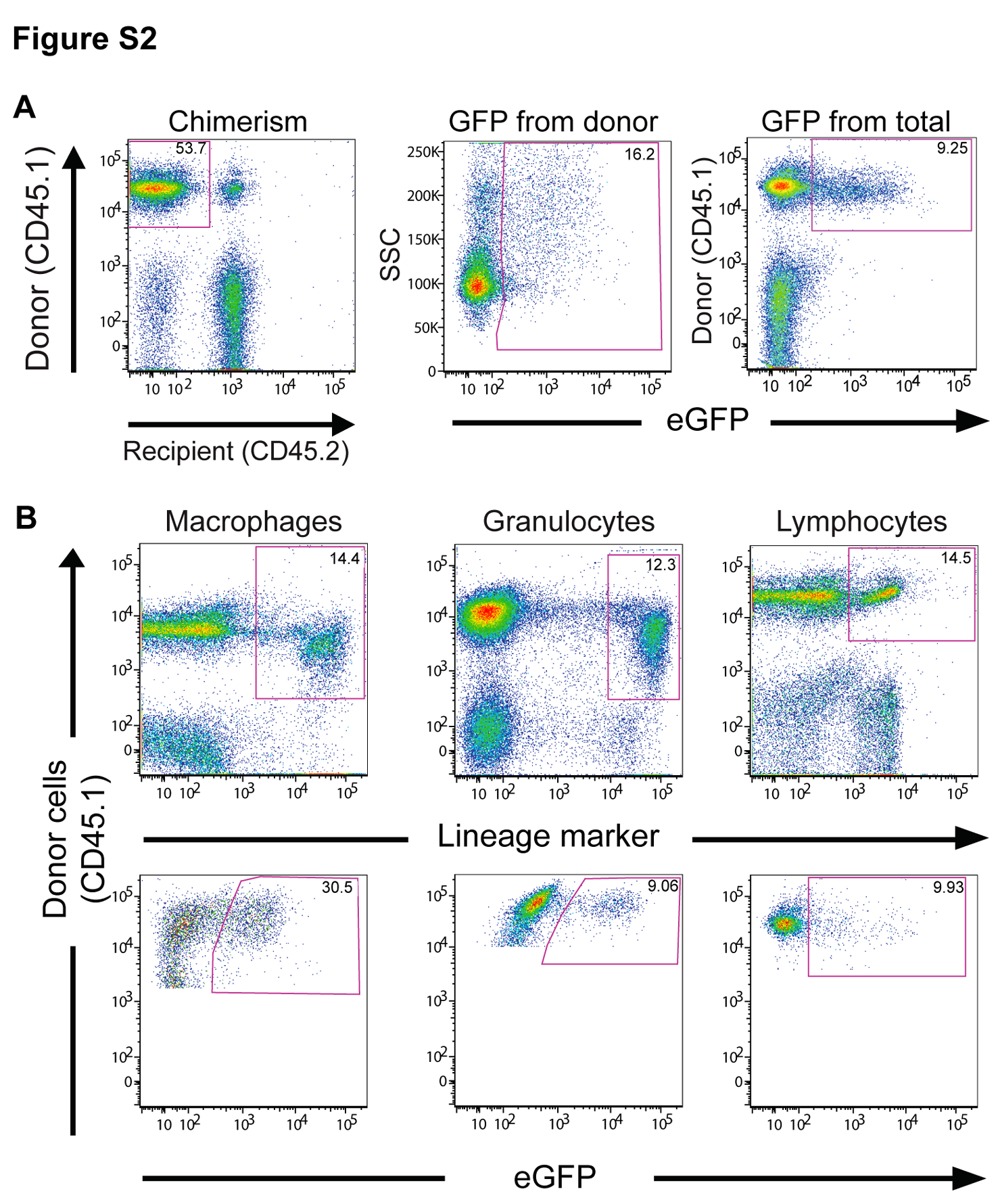

Supplement: Figure S2 — Chimerism, transduction and multilineage in vivo engraftment analysis of HSCs. A) Flow cytometry of peripheral blood from recipient mice 7 weeks after BMT plus one week with doxycycline treatment. Donor KGF-eGFP expressing blood cells were detected within the donor bone marrow population labelled with CD45.1 in the presence of doxycycline. B) Multilineage engraftment analysis by flow cytometry. Blood markers for macrophages (Mac-1), granulocytes (Gr-1) and lymphocytes (CD-3) were used together with the donor marker (CD45.1). In each differentiated subpopulation, KGF-eGFP expressing cells were detected, demonstrating an efficient bone marrow repopulation and in vivo induction. (7.89 MB TIF) [file pone.0008013.s002.tif]

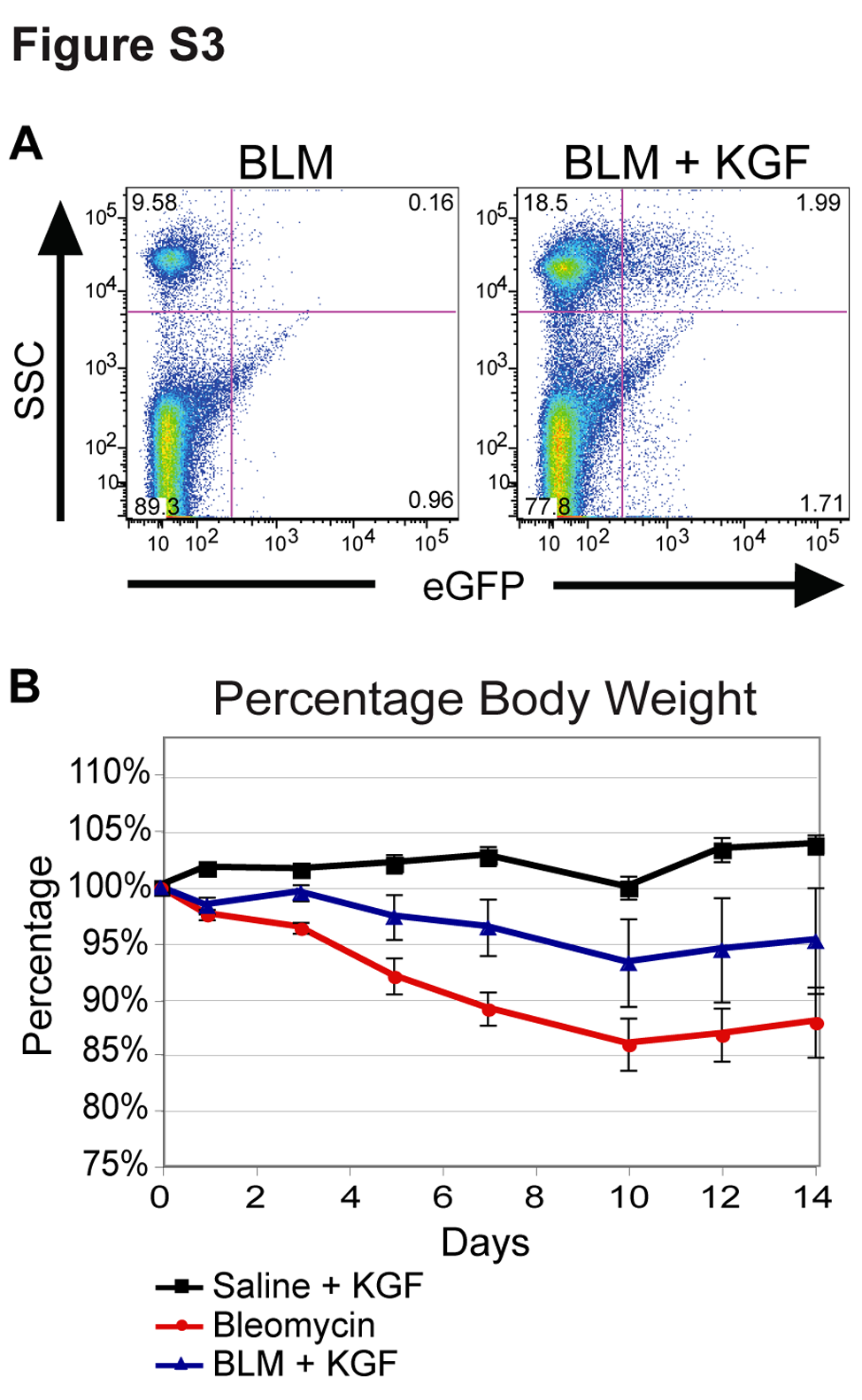

Supplement: Figure S3 — Expression of KGF-GFP in the lungs reduces body weight loss in mice after bleomycin administration. A) Flow cytometry of left lung digests detecting KGF-eGFP expressing cells 14 days after bleomycin administration. B) Body weights post bleomycin treatment showed doxycycline-induced expression of KGF from BMDC (BLM+KGF) reduces the weight loss induced by bleomycin. (4.63 MB TIF) [file pone.0008013.s003.tif]

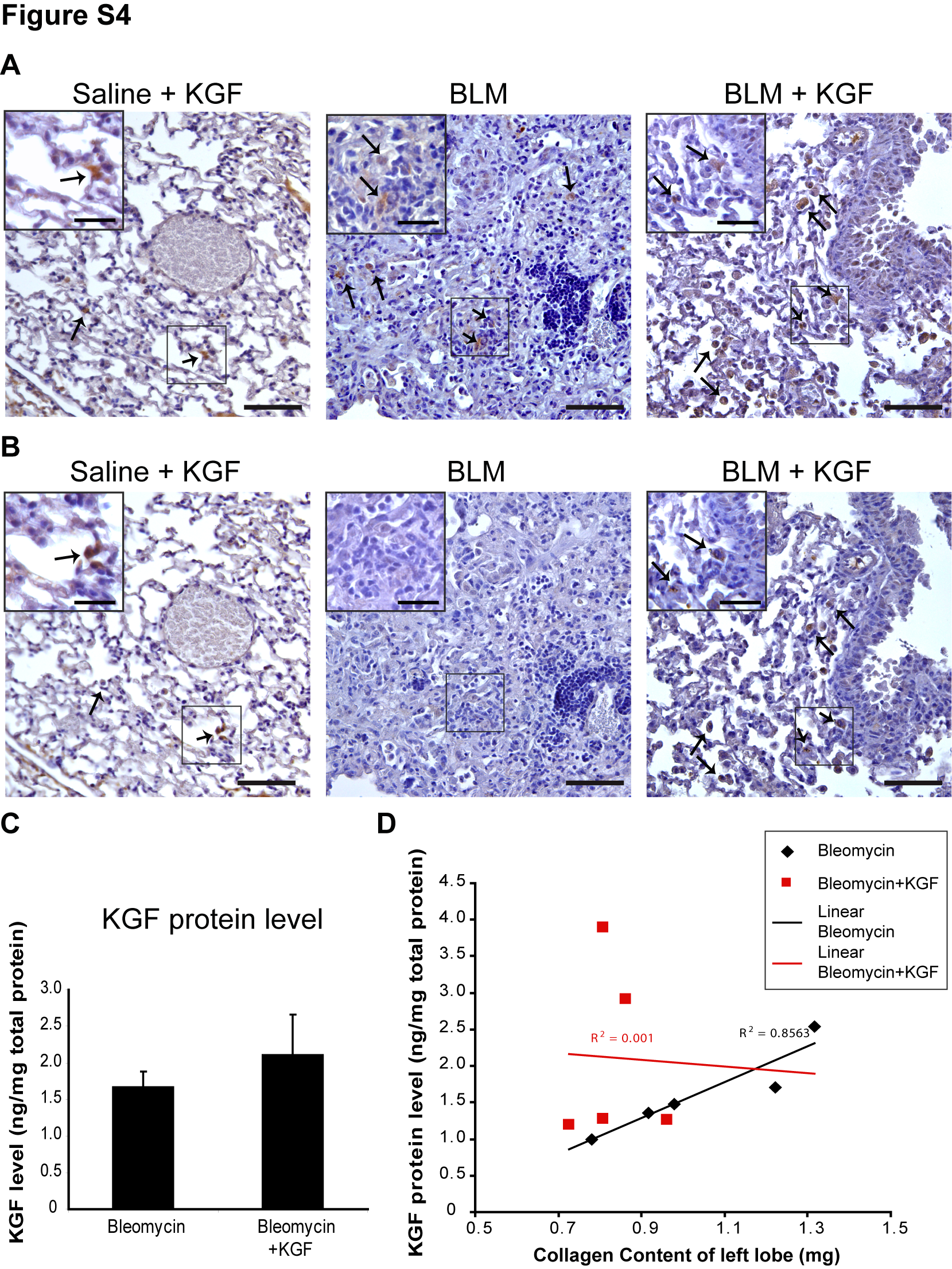

Supplement: Figure S4 — Increased detection of KGF positive cells in mice expressing HSC-KGF correlates with GFP positive cells. A) KGF immuno-staining 14 days after bleomycin demonstrated frequent brown stained cells (arrows) in mice treated with doxycyline. (Scale bars = 50 um; inserts, scale bars = 25 um). B) GFP staining in serial lung sections showed a correlation of KGF and GFP expression in mice treated with doxycyline only. (Scale bar = 50 um, inserts, scale bars = 25 um). C) KGF ELISA from lung powder showed higher levels of KGF in mice treated with doxycyline. D) Correlation of KGF protein levels, measured by ELISA, with the collagen content of the lungs after bleomycin injury. Linear correlation values are indicated for each group. (6.03 MB TIF) [file pone.0008013.s004.tif]
